# The central odontogenic fibroma: How difficult can be making a preliminary diagnosis

**DOI:** 10.4317/jced.52795

**Published:** 2016-04-01

**Authors:** Roberto Pippi, Marcello Santoro, Romeo Patini

**Affiliations:** 1 Department of Oral and Maxillofacial Sciences, “Sapienza” - University of Rome, Caserta street, 6 - 00161 Rome, Italy; 2 Department of Surgical sciences for head and neck diseases, School of dentistry, Catholic University of Sacred Heart, Largo A. Gemelli, 1 - 00168 Rome, Italy

## Abstract

Central odontogenic fibroma (COF) is a rare benign odontogenic tumor derived from the dental ectomesenchymal tissues. A 16-year-old Caucasian female patient was referred by her dentist for a radiolucent asymptomatic area associated with the crown of the impacted lower right third molar. A preliminary diagnosis of a follicular cyst was supposed. The lesion was surgically removed under general anesthesia together with the impacted tooth. The microscopic diagnosis of the excised tissue revealed an odontogenic fibroma. No clinical or radiographic signs of recurrence were found five years after surgical excision. Despite the various differential diagnoses of homogeneous unilocular and well delimited radiolucencies of the jaws, enucleation with peripheral curettage, without any other pre-operative imaging exams or biopsies, can be considered as the treatment of choice.

** Key words:**Differential diagnosis, impacted third molar, radiographic imaging, microscopic diagnosis, odontogenic fibroma.

## Introduction

The central odontogenic fibroma (COF) is an uncommon odontogenic tumor whose typing is controversial both clinically and histologically (1). COF is reported to occur in the 4-80 year range of age (mean age: 40 years) with a 2.2:1 female predilection. It accounts for 0.1-1.5% of all odontogenic tumors and 6.1% if odontoma is excluded. Approximately 55% of the cases occur in the mandible, half of which posterior to the first molar and up to one-third in conjunction with an un-erupted third molar (2). From a topographic point of view two different kinds of COF exist, an intra-osseous or central form and an extra-osseous or peripheral form (3). A recent report demonstrated that age distribution among central lesions showed a shallow curve, with all decades represented whereas the peripherally located lesions showed a predilection for the 2nd to 4th decades of life and that intraosseous tumors were relatively evenly distributed in the anterior, premolar and molar regions whilst peripheral lesions tended to arise in the anterior sextants of the jaws (4).

In 2005, WHO (5) defined two different histological types of COF, the simplex type, with only few epithelial isles, and the complex type, rich of epithelial cells. Because the presence of epithelium is a requisite to confirm the diagnosis, immunohistochemistry has been demonstrated to be helpful in epithelium-poor cases (6). The clinical and radiographic diagnosis of COF has been reported not to be always easy and the tumor should be differentiated from many other lesions of the jaws such as keratocystic odontontogenic tumor, ameloblastoma, odontogenic myxoma, ameloblastic fibroma, calcifying odontogenic cyst, dentigerous cyst since it could appear as a well-defined radio-transparent area associated with the crown of an impacted tooth.

The aim of this case report is to describe a case of COF in which the preliminary diagnosis was particularly difficult because the lesion mimicked a dentigerous cyst.

## Case Report

**Figure 1:**
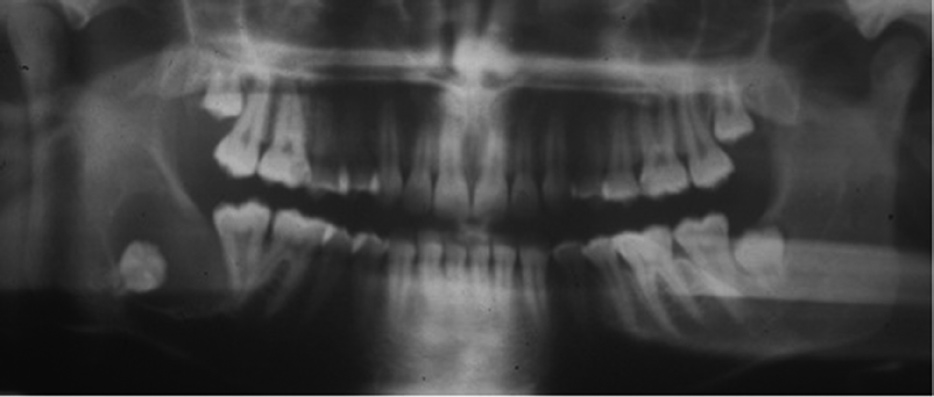
Pre-operative orhopantomography: a radiolucent area, with a quite evident radiopaque border, is detectable in the right lower third molar region, starting from the impacted third molar tooth collar. The second molar appears distally inclined.

A 16-year-old woman was referred by her dentist to the Oral Surgery Unit – Department of Oral and Maxillofacial Sciences, Faculty of Medicine and Dentistry, “Sapienza” University of Rome for a radiolucent area associated with the impacted right third molar whose radiographic characteristics mimicked those of a dentigerous cyst. The intraoral examination revealed no alterations, the gingival surface distal to the lower right second molar was healthy and smooth. The orthopantomography showed a radiolucent lesion located in the right mandibular angle containing an impacted right lower third molar (Fig. 1). A preliminary diagnosis of a dentigerous cyst was therefore supposed.

The surgery was conducted under general anesthesia. After a muco-periosteal flap was incised and raised, the ostectomy was performed to reach the pathologic tissue which was not adherent to the impacted tooth and therefore easily separated from it. Due to the fibrous aspect of the lesion, an odontogenic fibroma was considered as a probable diagnosis so that a thorough 1-2 mm curettage of the residual bone cavity was therefore performed after the complete excision of the pathological tissue and the impacted tooth removal.

**Figure 2:**
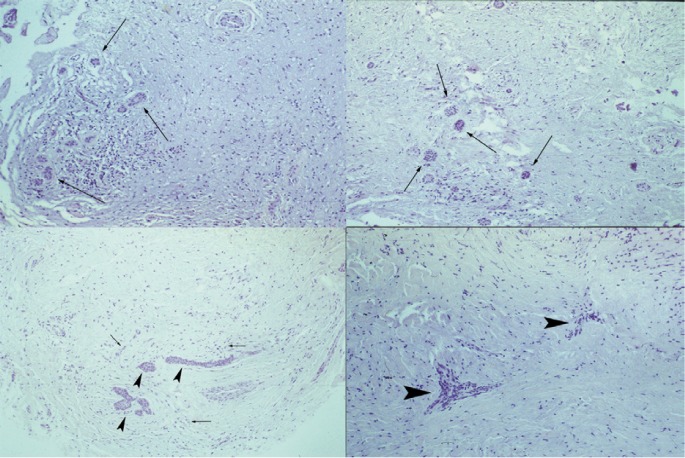
Histologic appearance (Hemat/Eos). 100x. Multiple areas of pathologic tissue consisting of connective tissue populated by distributed basophilic fibroblasts (arrows) and solid epithelial nests (arrowheads) scattered within this background.

**Figure 3:**
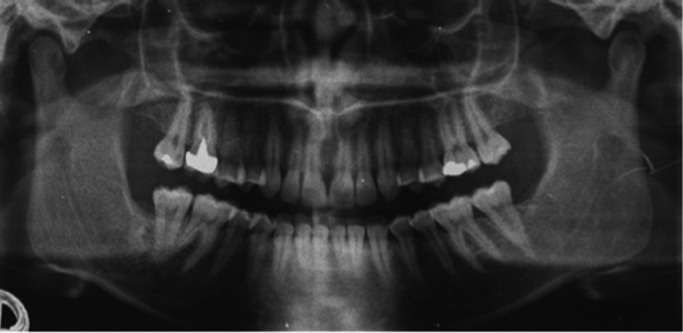
Five years orthopantomographic follow-up. The second molar seems slightly aligned.

The excised lesion was constituted by multiple fragments not exceeding 2,5 cm in diameter. All fragments were fixed with 10% buffered formalin and microscopically analyzed. Microscopic examination showed a cellular loose connective tissue with epithelial cell aggregates showing the typical morphological characteristics of the enamel organ. These aspects were compatible with the diagnosis of the simple type of odontogenic fibroma (Fig. 2). No recurrence was seen at the 5 years clinical and radiographic follow-up (Fig. 3).

## Discussion

The central odontogenic fibroma is an uncommon tumor which clinically appears as an asymptomatic well-defined osteolytic lesion and which rarely can be locally aggressive, with dental displacement and rhizolysis. Radiologically, COF appears as an uni- or multi-locular radiolucent area and it can be indistinguishable from other radio-transparent lesions making the pre-operative diagnosis more difficult. Actually, in the present case a dentigerous cyst was suspected from the two-dimensional x-ray performed.

Nevertheless the use of 3D x-ray exams, such as traditional CT or Cone Beam CT and high resolution nuclear magnetic resonance (HRNMR), could be considered to better investigate all jaw radiolucencies and correctly plan the surgery (7,8), thus resulting in a lower risk of contiguous anatomic structure surgical injury.

A correct pre-operative evaluation deeply influences the surgical approach in that a previous biopsy is highly indicated for tumors but not for cysts. If a benign tumor is diagnosed, a more extensive ostectomy and a more accurate curettage of the residual bone cavity should be performed in order to completely excise the lesion and to avoid recurrences (2). In this light, HRNMR or contrast-CT are nowadays indicated for pre-operative evaluation of multi-locular or/and ill-defined radiolucent lesions while direct enucleation with peripheral curettage, without any pre-operative imaging exams or biopsies, can be considered for uni-locular homogeneous radio-transparent and well-delimited lesions.

Due to the very low incidence of recurrence and the benign biological behavior of the COF, the surgical approach is usually conservative but, if a microscopic diagnosis has not been performed from a pre-operative biopsy, a more aggressive lesion, such as a keratocystic tumor or a unicystic ameloblastoma, that have a higher risk of recurrence in relation to their different pattern of local aggressiveness resulting in a longer follow-up required, cannot be excluded from the definitive diagnosis (2).

## Conclusions

The great variability in radiological appearance of the COF means that it should be considered in the differential diagnosis of all jaw radiolucencies. The case presented here shows how difficult can be making a preliminary diagnosis of COF although the intra-operative appearance of the lesion is suggestive and the prognosis is anyhow good, provided that a peripheral 1-2 mm curettage is performed.
